# Conjugal plasmid transfer in the plant rhizosphere in the One Health context

**DOI:** 10.3389/fmicb.2024.1457854

**Published:** 2024-08-29

**Authors:** Francesco Riva, Arnaud Dechesne, Ester M. Eckert, Valentina Riva, Sara Borin, Francesca Mapelli, Barth F. Smets, Elena Crotti

**Affiliations:** ^1^Department of Food, Environmental and Nutritional Sciences (DeFENS), University of Milan, Milan, Italy; ^2^Department of Environmental and Resource Engineering, Technical University of Denmark, Kongens Lyngby, Denmark; ^3^CNR – IRSA Water Research Institute, Molecular Ecology Group (MEG), Verbania, Italy; ^4^National Biodiversity Future Center, Palermo, Italy; ^5^Department of Biological and Chemical Engineering, Center for Water Technology, Aarhus University, Aarhus, Denmark

**Keywords:** horizontal gene transfer, conjugation, rhizosphere, endosphere, agri-food system, ready-to-eat vegetables, microbial community

## Abstract

**Introduction:**

Horizontal gene transfer (HGT) of antibiotic resistance genes (ARGs) is one of the primary routes of antimicrobial resistance (AMR) dissemination. In the One Health context, tracking the spread of mobile genetic elements (MGEs) carrying ARGs in agri-food ecosystems is pivotal in understanding AMR diffusion and estimating potential risks for human health. So far, little attention has been devoted to plant niches; hence, this study aimed to evaluate the conjugal transfer of ARGs to the bacterial community associated with the plant rhizosphere, a hotspot for microbial abundance and activity in the soil. We simulated a source of AMR determinants that could enter the food chain via plants through irrigation.

**Methods:**

Among the bacterial strains isolated from treated wastewater, the strain *Klebsiella variicola* EEF15 was selected as an ARG donor because of the relevance of *Enterobacteriaceae* in the AMR context and the One Health framework. The strain ability to recolonize lettuce, chosen as a model for vegetables that were consumed raw, was assessed by a rifampicin resistant mutant. *K. variicola* EEF15 was genetically manipulated to track the conjugal transfer of the broad host range plasmid pKJK5 containing a fluorescent marker gene to the natural rhizosphere microbiome obtained from lettuce plants. Transconjugants were sorted by fluorescent protein expression and identified through 16S rRNA gene amplicon sequencing.

**Results and discussion:**

*K. variicola* EEF15 was able to colonize the lettuce rhizosphere and inhabit its leaf endosphere 7 days past bacterial administration. Fluorescence stereomicroscopy revealed plasmid transfer at a frequency of 10^−3^; cell sorting allowed the selection of the transconjugants. The conjugation rates and the strain’s ability to colonize the plant rhizosphere and leaf endosphere make strain EEF15::*lacI^q^-pLpp-mCherry-gm^R^* with pKJK5::Plac::*gfp* an interesting candidate to study ARG spread in the agri-food ecosystem. Future studies taking advantage of additional environmental donor strains could provide a comprehensive snapshot of AMR spread in the One Health context.

## 1 Introduction

Horizontal gene transfer (HGT) is an evolutionary process that confers new phenotypes and metabolic activities to bacteria, allowing their rapid adaptation to new environmental selective pressures such as antibiotic presence (Aminov, 2011; Saak et al., 2020). Through various HGT mechanisms, mobile genetic elements (MGEs) harboring antibiotic-resistant genes (ARGs) can spread among microorganisms (Smalla et al., 2018). The diffusion of antimicrobial resistance (AMR) has been recognized as one of the serious global challenges of this century; as a consequence, treatments against bacterial infections are gradually losing efficacy, representing important threats to human health (Pinilla-Redondo et al., 2018; Larsson and Flach, 2022). Thus, it is crucial to track how AMR determinants can move—individually and collectively—among environmental bacterial communities and to understand how their transfer is influenced by the complexity of the ecosystems (Saak et al., 2020).

In the One Health perspective, studies on the spread of AMR determinants should include the agri-food ecosystems. The presence of AMR determinants has been documented in specific hotspots of the agri-food and related ecosystems, such as managed fields, rhizosphere, and wastewater treatment plants (WWTPs) (Larsson and Flach, 2022; Riva V. et al., 2020). Treated wastewater, in particular, can convey and promote the maintenance of an antibiotic resistome in freshwater habitats (Corno et al., 2019; Di Cesare et al., 2015), and, through the reuse of treated wastewater for irrigation, ARGs and antibiotic-resistant bacteria (ARB) might enter the food chain via plants (Riva F. et al., 2020; Riva et al., 2022a; Musiyiwa et al., 2024). ARB and ARGs have indeed been found in fresh products and farm environments, underlining the risks to which humans are exposed (Perera et al., 2020; Araújo et al., 2017; Yang et al., 2020; Nüesch-Inderbinen et al., 2015; Schierstaedt et al., 2019; Cerqueira et al., 2019).

Among HGT mechanisms, conjugation usually takes place in environments with high cell density and high metabolic activity (Ulrich et al., 2015), i.e., where high density of cells can increase chance for contacts (Riva V. et al., 2020), such as rhizosphere, mycelia, sewage sludge and host-associated microbial communities (Van Elsas et al., 2003; Qiu et al., 2018; Berthold et al., 2016; Virolle et al., 2020). Conjugation is influenced by different factors, e.g., temperature, nutrient ability, and cell-to-cell contact (Zarei-Baygi and Smith, 2020), as well as the presence of emerging pollutants, which could enhance the spread of ARGs (Feng et al., 2021; Yu et al., 2021). The transfer can occur between bacteria from the same species or between taxonomically distant ones (Zarei-Baygi and Smith, 2020; Klümper et al., 2015); for instance, the taxonomic relatedness between donors belonging to *Escherichia coli* species and recipient bacteria has been recently investigated in a meta-analysis and found to influence conjugal rate in liquid conditions, but not on solid surfaces (Alderliesten et al., 2020). The genetic background of *E. coli* donor strains has also been shown to affect the conjugal transfer of the broad host range IncP-1 plasmid pKJK10 when transferred to a certain *E. coli* recipient strain in genome-wide association studies, which encompassed deletions, insertions, recombinations, as well as the presence or absence of genes (Van Wonterghem et al., 2022). Focusing on the identification of genes involved in conjugation, authors have, indeed, found several chromosomally encoded genes of *E. coli* that positively impact transfer, i.e., genes *fliF* and *fliK* involved in flagella assembly, *ucpA* expressing an oxidoreductase, and *kefB* encoding a potassium transporter (Van Wonterghem et al., 2022).

Several approaches have been developed to track conjugation events in specific environmental niches, such as soil (Klümper et al., 2015; Fan et al., 2019) or sewage treatment plant communities (Li et al., 2020). The rhizosphere is the portion of soil strictly attached to the plant roots and influenced by root exudates. Because of its high content of nutrients, as well as its bacterial abundance and mobility, it has been indicated as a hotspot of HGT, especially via conjugation (Van Elsas et al., 2003). However, ARG transfer in the rhizosphere and other plant compartments has been poorly investigated so far (Sánchez-Salazar et al., 2023; Xu et al., 2021; Musiyiwa et al., 2024), but this knowledge is necessary to understand the magnitude of AMR diffusion, also considering how microbiomes respond to changing environmental conditions (Smalla et al., 2018; Pinilla-Redondo et al., 2018; Larsson et al., 2018; Saak et al., 2020; Abs et al., 2023). Thus, this study aimed to evaluate the conjugal transfer of plasmids within the rhizosphere microbiome, taking advantage of a bacterial donor strain of environmental origin. We focused our attention on the *Enterobacteriaceae* family because of its relevance in the One Health context: this family is known to include both pathogenic and commensal bacteria and can colonize several environments linked to the agri-food system, e.g., soil, plant niches, freshwater bodies, and human and animal gut (Touchon et al., 2020; Riva F. et al., 2020; Leimbach et al., 2013; Nüesch-Inderbinen et al., 2015; Wang et al., 2020; Schikora et al., 2012; Zhang et al., 2020). Specifically, we selected an *Enterobacteriaceae* strain isolated from the effluent from a municipal WWTP to mimic a real scenario of ARB released by WWTP and entering the agri-food system. The selected strain was genetically modified and used as an environmental donor strain to study ARG spread to the rhizosphere bacterial community associated with lettuce, chosen as a model of vegetables eaten raw. The results of conjugation experiments were then compared to the ones obtained with a strain of laboratory origin belonging to the *Enterobacteriaceae* family (i.e., *E. coli* MG1655).

## 2 Materials and methods

### 2.1 Bacteria isolation and identification

A sample of 1.1L of water was collected from the effluent from the largest municipal WWTP of Milan municipality (Milano-Nosedo, Northern Italy) and filtered through a 0.22-μm membrane filter GSWP04700 by vacuum pump. Bacteria on the filter were resuspended in saline solution (0.9% NaCl), serially diluted, plated on Violet Red Bile Lactose Agar (Thermo Fisher Scientific) supplemented with 100 μg/mL cycloheximide and incubated at 30°C for 48 h. Single colonies were randomly picked and spread 3 times on the same medium to obtain pure cultures. Pure cultures were stored at −80°C in 20% glycerol stocks. Genomic DNA was extracted from strains by boiling cell lysis (Ferjani et al., 2015) and the bacteria were identified by 16S rRNA gene amplification and partial sequencing followed by basic local alignment search tool (BLAST) alignment on the NCBI database (Callegari et al., 2020).

### 2.2 Antibiotic sensitivity

The bacterial collection was screened for strains sensitivity to kanamycin (100 μg/mL) and rifampicin (RIF) (100 μg/mL). Briefly, isolates’ growth was checked on Luria Bertani agar plates supplemented with the aforementioned antibiotics, after an incubation of 72 h at 30°C. *Klebsiella variicola* EEF15, selected as the donor strain, was also screened for sensitivity against gentamycin (100 μg/mL), as reported by Klümper et al. (2015).

### 2.3 Lettuce plants and agricultural soils

Experiments were conducted with two different setups. Colonization experiments in the lettuce rhizosphere were carried out with *Lactuca sativa* seedlings transplanted in agricultural soil collected in Triuggio (Monza and Brianza, Italy). In contrast, filter mating conjugation experiments between the donor strain and the lettuce rhizosphere community were carried out with *L. sativa* seedlings transplanted in agricultural soil collected from CRUCIAL (closing the rural–urban nutrient cycle) agricultural field site (Taastrup, Denmark), from a plot fertilized with NPK (Fernández-Calviño et al., 2021; Bollmann et al., 2017; Fernández-Calviño et al., 2017). Experiments were performed at least 1 week after the transplantation of lettuce plants from pot soil to agricultural soil. Lettuce plants were grown as described by Riva F. et al. (2020).

### 2.4 Root and leaf endosphere colonization by *Klebsiella variicola* EEF15 RIF-R

A rifampicin-resistant (RIF-R) mutant of the strain *K. variicola* EEF15, isolated from the effluent of the WWTP, was obtained as described in Riva F. et al. (2020) and used to test its ability to colonize the lettuce rhizosphere. Bacterial suspensions were grown overnight growth, and the following day, they were prepared to bacterize plant roots with a final concentration of 1 × 10^8^ and 1 × 10^9^ cells of EEF15 RIF-R per gram of soil (indicatively 300 g of soil were added per pot). Plant bacterization was performed by applying the bacterial suspensions to the soil surrounding the collar of potted lettuce plants (*n* = 3). After 1 week, the rhizosphere was collected (Marasco et al., 2018), and EEF15 RIF-R colonization was assessed by plating different serial dilutions of the rhizosphere samples on Luria broth (LB) medium supplemented with 100 μg/mL RIF in triplicate (Riva F. et al., 2020). The identity of randomly selected RIF-R colonies (*n* = 18) was confirmed by an intergenic transcribed spacer (ITS)-polymerase chain reaction (PCR) fingerprinting, comparing ITS-PCR profiles with the one of the EEF15 RIF-R strain (Mapelli et al., 2013).

*K. variicola* EEF15 RIF-R to colonize leaf endosphere, lettuce plants (*n* = 3) were bacterized with 1 × 10^9^ cells of EEF15 RIF-R per gram of commercial soil. After 1 week, the leaf endosphere microbiome was extracted as per Klerks et al. (2007). Approximately 10 g of lettuce leaves per plant (sampling both grown and newly formed leaves) were washed twice for 3 min in sterile distilled water, followed by a wash for 30 s with a solution of ethanol (70%), and finally washed for 3 times for 3 min in sterile distilled water. After the washing procedures, lettuce leaves were smashed in 10 mL of distilled water. The extract from the leaves was subsequently diluted and plated on LB and added with RIF (100 μg/mL). The distilled water used during the last washing procedure was plated on LB Petri dishes with RIF (100 μg/mL) to verify the sterilization of lettuce leaves. As per the rhizosphere, the identity of randomly selected RIF-R colonies (*n* = 12) was confirmed by ITS-PCR fingerprinting (Mapelli et al., 2013).

### 2.5 Donor strain construction

The strain *K. variicola* EEF15 was chromosomally tagged with a cassette containing a *mCherry* gene, a constitutively expressed *lacI^q^* gene, and a gentamycin resistance gene (*gm^R^*); the broad host range plasmid pKJK5::Plac::*gfp*, carrying a green fluorescent protein gene under the control of *lacl^q^* gene repressible promoter and a kanamycin resistance gene (*km^R^*), was then introduced into the strain. Insertion of the gene cassette on the chromosome and the consequent loss of the plasmid used to insert the gene cassette was carried out as previously described (McKenzie and Craig, 2006; Fournet-Fayard et al., 1995) and verified by fluorescence microscopy and PCR analysis (Lambertsen et al., 2004). Briefly, the suicide plasmid pGRG36 containing the *lacI^q^-pLpp-mCherry-gm^R^* cassette was inserted by electroporation at 1800 V (Fournet-Fayard et al., 1995) and transformed cells were selected on LB agar plates with gentamicin (100 μg/mL). As explained by Mckenzie and Craig (2006), to ensure the complete loss of the plasmid, red fluorescent colonies grown on the selective media were streaked twice and incubated at 42°C. Primers used to verify the loss of the plasmid were (i) Backbone pGRG36Fw 1 5′-TAG AGC GTC GCT ATT GGC AG-3′; Backbone pGRG36Rv 1 5′-TGC TCA ACG AGT TCG CTT CT-3′ and (ii) Backbone pGRG36Fw 8 5′-TGC TAG AGG CAT TAC GCT CG-3′; Backbone pGRG36Rv 8 5′-GCA AAG CGG GCA AAT ACC AA-3′. Primers were designed using the Primer designing tool—National Center for Biotechnology Information (NCBI).^1^ Insertion of the plasmid pKJK5::Plac::*gfp* in electrocompetent cells was carried out by electroporation at 1800 V (Fournet-Fayard et al., 1995); transformed cells were selected on LB agar plates with kanamycin (100 μg/mL). The acquisition of the plasmid carrying the *gfp* was verified by fluorescence microscopy (*gfp* expression), adding 0.1 μM IPTG in the growth media. The strain EEF15::*lacI^q^-pLpp-mCherry-gm^R^* with plasmid pKJK5::Plac::*gfp* was then used in conjugation experiments.

### 2.6 Conjugal transfer to the selected recipient strain *Pseudomonas putida* KT2440

Conjugation assays were performed using filter mating assays between the donor strain *K. variicola* EEF15::*lacI^q^-pLpp-mCherry-gm^R^* with pKJK5::Plac::*gfp* and the recipient *P. putida* KT2440 (Schlechter and Remus-Emsermann, 2019). Bacteria were grown separately in LB liquid media, and 10^9^ cells/ml of each strain (calculated by using a Thoma cell-counting chamber) were mixed and inoculated on a nitrocellulose membrane filter (GSWP, 25-mm diameter, 0.22 μm pore size, Millipore, Burlington, MA, USA) placed on LB agar plates. The filters were incubated for 24 h at 30°C. Transconjugants were selected by plating the conjugation mix on cetrimide agar (Millipore) added with kanamycin (100 μg/mL): donor growth was inhibited on cetrimide agar. In contrast, the growth of *P. putida* KT2440 without the conjugative plasmid was inhibited by the presence of kanamycin. Transfer of the plasmid in recipient cells was verified by fluorescent microscopy (monitoring the *gfp* expression) and PCR amplification (of the *gfp* gene; Riva et al., 2022a), and by carrying out ITS-PCR amplification to confirm strain identification (Mapelli et al., 2013).

### 2.7 Conjugal transfer to the bacterial community of lettuce rhizosphere

Rhizosphere was collected from lettuce plants as described by Marasco et al. (2018). Briefly, lettuce roots were collected in a 50-ml tube, immersed in physiological water, and vortexed to remove the attached rhizosphere; roots were then separated from the rhizosphere and the rhizosphere was used for conjugation assays. Conjugation between strain EEF15::*lacI^q^-pLpp-mCherry-gm^R^* tagged with plasmid pKJK5::Plac::*gfp* and the lettuce rhizosphere community was conducted as already performed by Klümper et al. (2014), modifying two steps of the protocol. In particular, (i) the microbiome of the rhizosphere was extracted as described by using the Nycodenz density gradient (Fan et al., 2019); and (ii) a cell density of 3.38 × 10^8^ cells/ml, instead of 3.38 × 10^7^ cells/ml, for the donor strains and the pool of recipient bacterial cells were used. Cell concentrations were estimated using a Thoma cell-counting chamber. To estimate the conjugation transfer rate of plasmid pKJK5::Plac::*gfp* from strain EEF15::*lacI^q^-pLpp-mCherry-gm^R^*, filter mating experiments were performed also using *E. coli* MG1655::*lacI^q^-pLpp-mCherry-km^R^* with plasmid pKJK5::Plac::*gfp*, as donor strain (Klümper et al., 2015; Supplementary Table 1). Filters placed on plates containing 10% soil extract medium were incubated at 25°C for a period of 48 h. The ability of the donor strains to transfer pKJK5::Plac::*gfp* plasmid to the rhizosphere microbiome was evaluated by fluorescence stereomicroscopy and calculated as conjugal transfer rate, as reported in the following formula: [Transconjugants per pictures × filter area (µm^2^)]/[picture area (µm^2^) × recipients introduced originally]. The number of transconjugants was estimated by counting the green microcolonies using fluorescence stereomicroscopy.

### 2.8 Sorting and identification of transconjugants

Transconjugants were sorted and identified as per Klümper et al. (2014). Briefly, bacteria on the filter were detached and resuspended in PBS solution, added with 30% glycerol, and stored at −80°C. After 4 weeks, the samples were thawed and sorted on a BD FACSAria. Transconjugants were selected by *gfp* expression, excluding non-fluorescent soil bacteria and all the background particles. DNA from the sorted samples was extracted using InstaGene™ Matrix (Bio-Rad); hypervariable regions V3-V4 were amplified, and the Illumina library was prepared as described in Li et al. (2021). Amplicons were further sequenced through the Illumina MiSeq platform using Reagents Kit v3 (2 × 300 cycles) in the paired-end mode at Copenhagen University.

### 2.9 Metataxonomic sequencing analyses

Reads were filtered, merged, and grouped in amplicon sequence variants (ASVs) with the DADA2 pipeline (Callahan et al., 2016; Callahan et al., 2017) as suggested in the online tutorial, except for filtering that was done with maxEE “2” for R1 and “10” for R2. Taxonomy was assigned using the Silva Database version 138 (Yilmaz et al., 2014), while the microbiome data were analyzed by phyloseq R package (McMurdie and Holmes, 2013). β-Diversity was calculated as weighted UniFrac Distance and plotted as principal coordinate analysis (PCoA). α-Diversity was calculated as Richness (number of ASVs), Shannon, and Simpson indices. To determine differences between the communities that were treated with the two donors, analysis of compositions of microbiomes with bias correction (ANCOMBC) using the ANCOMBC package in R was conducted for both the ASV table and genera table, and the results were inspected for differentially distributed taxa (Lin and Peddada, 2020). All analyses were performed using RStudio.^2^ Sequences affiliated with the *Klebsiella* and *Escherichia* genera (6.5% of all reads) were removed from the amplicon metagenomics sequencing dataset to eliminate any possible contamination due to their presence in sorted transconjugants. These genera were also removed from the other samples and the control to not create artificial differences between the treatments, even though this can cause the loss of some transconjugants from the results.

## 3 Results

### 3.1 Donor strain isolation, selection, and construction

The selective medium Violet Red Bile Lactose Agar was used to obtain a collection of *Enterobacteriaceae* strains from the effluent of a municipal WWTP. A total number of 14 isolates, including 9 *Klebsiella*, 2 *Enterobacter*, 1 *Citrobacter*, 1 *Kluyvera*, and 1 *Raoultella*, were purified and identified by 16S rRNA gene sequencing (Table 1). All the isolates showed sensitivity against kanamycin and RIF (Table 1). Specifically, RIF sensitivity was investigated to further select a RIF-sensitive strain to obtain a RIF-resistant (RIF-R) mutant to be used for plant colonization experiments; sensitivity against kanamycin was instead tested since needed for the construction of the donor strain (the plasmid pKJK5::Plac::*gfp* carries a *km^R^* gene). Among RIF- and kanamycin-sensitive isolates, the strain *K. variicola* EEF15 was selected as a donor for conjugation experiments because the *Klebsiella* genus resulted in the most representative one among the obtained isolates. The strain EEF15 was genetically modified on the chromosome by adding a cassette carrying a red fluorescent protein (mCherry), a *lacI^q^* repressor gene, and a gentamycin resistance gene (*gm^R^*). Finally, the plasmid pKJK5::Plac::*gfp* carrying a g*fp* gene, the expression of which was under the control of *lacI^q^* gene carried on the chromosome, was introduced in the strain. The resulting mutant was gentamycin-resistant and expressed red fluorescence. The gene *gfp*, plasmid encoded, would be only expressed in a recipient strain of the rhizosphere microbiome missing *lacI^q^* gene. The strategy we used to evaluate HGT in the rhizosphere microbiome consisted of two steps: initially, we extracted the cells of the microbial community associated with the lettuce rhizosphere by using a density gradient, and we then exposed it to the donor strain obtained as previously described (Supplementary Figure 1).

**Table 1:** List of strains isolated from the effluent of the wastewater treatment plant (WWTP) of Nosedo (Milan, Italy).

Strain	Closest species	Accession number of the closest species	% (bp/bp)	Kanamycin resistance	Rifampicin (RIF) resistance
EEF1	*Enterobacter cloacae*	MK649836	99% (1393/1394)	−	−
EEF3	*Kluyvera georgiana*	NR_024883	99% (1382/1390)	−	−
EEF4	*Klebsiella pasteurii*	MN104667	99% (1204/1209)	−	−
EEF5	*Citrobacter braakii*	KR149005	99% (1385/1389)	−	−
EEF6	*Klebsiella michiganensis*	MG571767	100% (1389/1389)	−	−
EEF7	*Klebsiella michiganensis*	CP033824	100% (1379/1379)	−	−
EEF8	*Klebsiella oxytoca*	MG576171	99% (1400/1402)	−	−
EEF9	*Klebsiella huaxenensis*	CP036175	99% (1394/1397)	−	−
EEF10	*Klebsiella pasteurii*	MN104667	99% (1395/1398)	−	−
EEF11	*Klebsiella michiganensis*	CP029770	99% (1394/1396)	−	−
EEF12	*Klebsiella michiganensis*	CP042545	99% (1378/1379)	−	−
EEF13	*Enterobacter cloacae*	LR607347	100% (1373/1373)	−	−
EEF14	*Raoultella ornithinolytica*	LC504037	99% (1379/1381)	−	−
EEF15	*Klebsiella variicola*	LR130544	99% (1390/1391)	−	−

Identification of the bacterial isolates by partial 16S rRNA gene sequencing: 16S rRNA identification and accession number of the closest relative species, and percentage of identity and length (bp/bp) of the nucleotide sequence are reported. In the last two columns, the characterization of the sensitivity/resistance to kanamycin and rifampicin (RIF) is included. −, sensitive; +, resistant.

### 3.2 Rhizosphere and endosphere colonization by the donor strain

**Figure 1:**
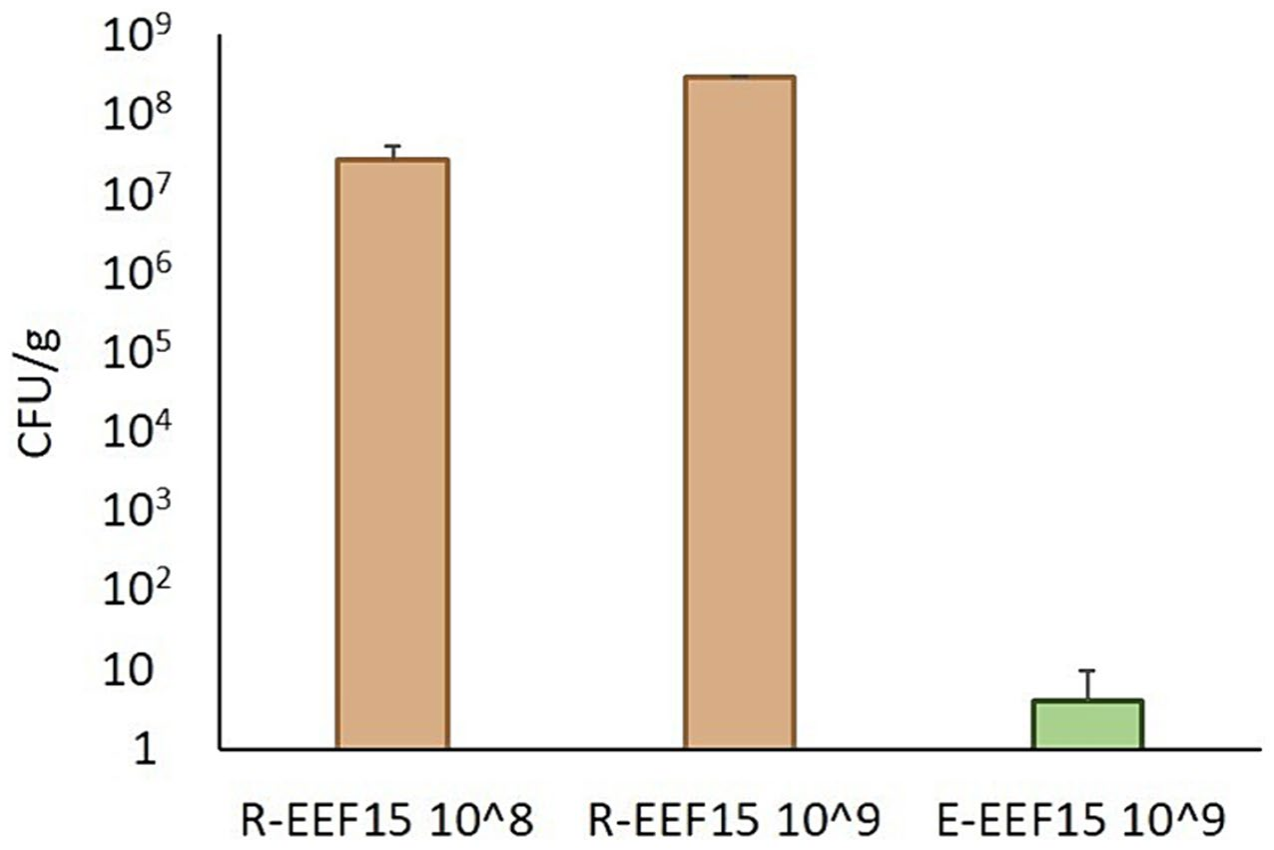
Lettuce colonization by *Klebsiella variicola* EEF15 RIF-R when lettuce rhizosphere was bacterized with 10^8^ and 10^9^ cells/g of soil (indicated as “10^8^” and “10^9^” in the sample names). EEF15 RIF-R abundance in the rhizosphere (“R” samples) and in the leaf endosphere (“E” sample) is indicated as CFU/g of soil or leaf tissue, respectively.

The ability of the EEF15 strain to colonize the lettuce rhizosphere and endosphere was verified using the RIF-R strain EEF15 RIF-R. This ability is indeed important to study the spread of AMR at the plant level, considering that an antibiotic-resistant strain could reach the plant due to the reuse of treated wastewater as an irrigation source. The results showed that 1 week after the bacterization of the lettuce rhizosphere, 2.62 × 10^7^ ± 1.21 × 10^7^ CFU/g of the soil of RIF-R bacteria were present in the rhizosphere when 1 × 10^8^ EEF15 RIF-R cells/g of soil were applied (Figure 1; Supplementary Table 2). Similarly, when 1 × 10^9^ EEF15 RIF-R cells/g of soil were used, 2.87 × 10^8^ ± 1.53 × 10^7^ CFU/g of soil of RIF-R bacteria were isolated from bacterized plant rhizosphere (Figure 1; Supplementary Table 2). No RIF-R CFUs were detected in the negative control (i.e., not bacterized lettuce plants). ITS-PCR fingerprinting obtained from rhizosphere colonization experiments confirmed their identity as EEF15 RIF-R (Supplementary Figure 2A). Seven days after the rhizosphere bacterization with 1 × 10^9^ cells/ml, EEF15 RIF-R was isolated from the leaf endosphere, resulting in an average of 4.12 CFU/g of leaves (Figure 1; Supplementary Table 2). No RIF-R CFUs were found in the negative control (i.e., not bacterized lettuce plants) and in the distilled water used for washing procedures. ITS-PCR fingerprinting on selected colonies, obtained from endosphere colonization experiments, confirmed their identity as EEF15 RIF-R (Supplementary Figure 2B).

### 3.3 Transfer of plasmid pKJK5::Plac::*gfp* to a known bacterial recipient

We first assessed the transfer of plasmid pKJK5::Plac::*gfp* from the donor *K. variicola* EEF15::*lacI^q^-pLpp-mCherry-gm^R^* to the strain *P. putida* KT2440, used as the recipient in previous studies (Berthold et al., 2016). Filter mating experiments, based on Schlechter and Remus-Emserman, 2019, were performed using a ratio between the donor and the recipient strains equal to 1:1. The identity of random kanamycin-resistant transconjugants colonies was verified by ITS-PCR fingerprinting, showing to be the same of the recipient *P. putida* KT2440. Plasmid acquisition by transconjugants was verified using fluorescence microscopy, antibiotic selection, and *gfp* amplification through PCR (Supplementary Figure 3A).

### 3.4 Conjugation with the bacterial community of lettuce rhizosphere

*Klebsiella variicola* EEF15::*lacI^q^-pLpp-mCherry-gm^R^* harboring the plasmid pKJK5::Plac::*gfp* was incubated with the natural bacterial community obtained from lettuce rhizosphere. Likely, also the strain *E. coli* MG1655::*lacI^q^-pLpp-mCherry-km^R^* harboring the plasmid pKJK5::Plac::*gfp* was used as a donor in conjugal experiments with the bacterial community obtained from the lettuce rhizosphere (Supplementary Table 1). Mating filters were analyzed using fluorescence stereomicroscopy, showing the presence of red- and green-tagged colonies, hence confirming both the presence of donors as well as the transfer of plasmid in bacterial recipients, respectively (Supplementary Figure 3B). Transfer frequencies from *Klebsiella* or *Escherichia* donor strains to the recipient community are listed in Table 2, while Supplementary Table 3 reports the number of transconjugants detected in each of the 20–21 analyzed portions for each filter. The conjugal transfer rate from *K. variicola* EEF15 donor strain, even if resulted similar and with the same order of magnitude (i.e., 2.41 × 10^−3^ ± 5.71 × 10^−4^ for *K. variicola* EEF15 and 3.50 × 10^−3^ ± 1.60 × 10^−4^ for *E. coli* MG1655), was significantly lower than the one from *E. coli* MG1655 (analysis of variance [ANOVA], *p* < 0.05). No green events (presence of transconjugants) were detected when only the donors or the recipient cells were placed separately on the filters.

**Table 2:** Transfer frequency of plasmid pKJK5::Plac::*gfp* between *Klebsiella variicola* EEF15::*lacI^q^-pLpp-mCherry-gm^R^* or *Escherichia coli* MG1655::*lacI^q^-pLpp-mCherry-km^R^*, and the bacterial community of lettuce rhizosphere, calculated per each filter.

Filter	Transfer frequency	Average transfer frequency (SD)
Filter EEF15 donor No. 1	2.48 × 10^−3^	2.41 × 10^−3^ ± 5.71 × 10^−4^
Filter EEF15 donor No. 2	2.43 × 10^−3^	
Filter EEF15 donor No. 3	1.66 × 10^−3^	
Filter EEF15 donor No. 4	3.05 × 10^−3^	
Filter MG1655 donor No. 1	3.30 × 10^−3^	3.50 × 10^−3^ ± 1.60 × 10^−4^
Filter MG1655 donor No. 2	3.69 × 10^−3^	
Filter MG1655 donor No. 3	3.51 × 10^−3^	
Filter MG1655 donor No. 4	3.52 × 10^−3^	

The average value and standard deviation (SD) are also reported for each treatment. SD, standard deviation.

**Figure 2:**
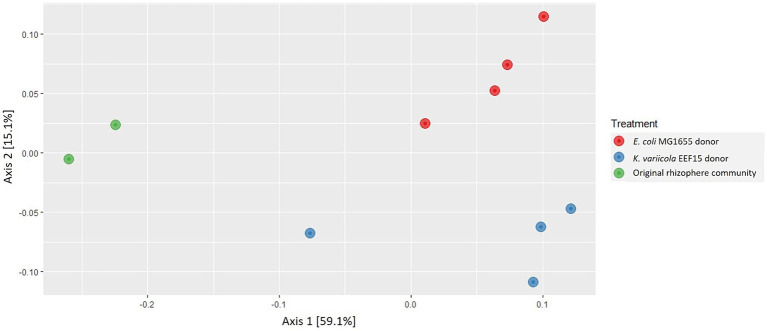
Multidimensional scaling (MDS)/principal coordinate analysis (PCoA) on weighted UniFrac Distance of the sample replicates of each treatment, i.e., transconjugants obtained using *Escherichia coli* MG1655::*lacI^q^-pLpp-mCherry-km^R^* donor strain, transconjugants obtained using *Klebsiella variicola* EEF15::*lacI^q^-pLpp-mCherry-gm^R^* donor strain and the original bacterial community of lettuce rhizosphere.

**Figure 3:**
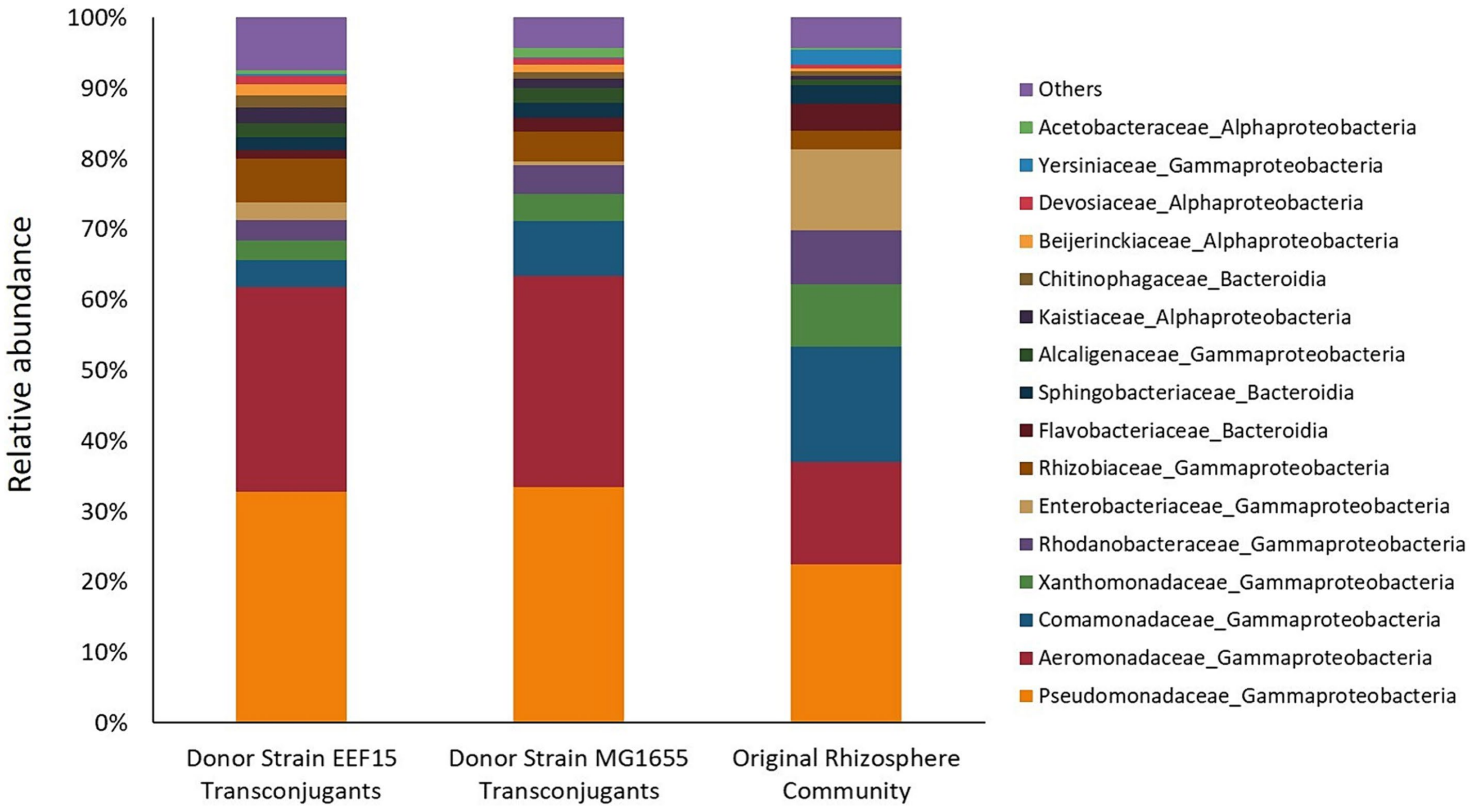
Relative abundance of ASVs related to filter mating assays and from the original rhizosphere bacterial community (higher than 0.05% for at least one treatment) at the family level. Eighty-two ASVs, whose relative abundance is lower than 0.05%, are classified as “Others.” From the left to the right, histograms refer to transconjugants obtained with *Klebsiella variicola* EEF15::*lacI^q^-pLpp-mCherry-gm^R^* and *Escherichia coli* MG1655::*lacI^q^-pLpp-mCherry-km^R^* donor strains. The last histogram showed the relative abundance of bacterial families of the bacterial community originally associated with the lettuce rhizosphere.

Following cell sorting and subsequent DNA extraction from sorted cells, the identity of transconjugants was assessed through 16S rRNA gene amplicon sequencing, retrieving a total number of 112,748 reads, corresponding to 1,280 ASVs (Supplementary Table 4). All the samples showed sufficient sequencing depth to capture taxa diversity (Supplementary Figure 4). According to ANOVA analysis, the α-diversity indices (Richness, Shannon, and Simpson) of the transconjugant bacterial populations did not show statistical differences depending on the bacterial donors used in the experiments (ANOVA, *p* > 0.05; Supplementary Table 5). β-Diversity was checked at ASV level: Multidimensional scaling (MDS)/PCoA on weighted UniFrac Distance is shown in Figure 2, showing a distinct clustering of the transconjugants compared with the original lettuce bacterial community. Among the transconjugants, a secondary grouping by the donor (*E. coli*/*K. variicola*) was revealed. ANCOMBC analysis showed that none of the ASVs or genera were significantly differently distributed between the communities derived from the two donors (data deposited in GitHub as indicated in the “Data Availability Statement” section). Figure 3 shows the average relative abundance of ASVs at the family level considering donor strains or original rhizosphere community (Supplementary Table 4), whereas Supplementary Figure 5 depicts the relative ASV abundance considering each sample. Eighty-two ASVs with a relative abundance lower than 0.05% for at least one treatment were classified as “Others.”

The results show that transconjugants were affiliated with the major bacterial phyla found in the original rhizosphere bacterial community. The most represented bacterial phylum was *Pseudomonadota*, with the *Gammaproteobacteria* class comprising approximately 60% of the transconjugants. Families of *Pseudomonadaceae* and *Aeromonadaceae* were the most abundant ones, representing, respectively, approximately 30 and 29% of the transconjugants. *Alphaproteobacteria* class represented 12.9 and 8.2% of the total ASVs retrieved in experiments with EEF15 and MG1655 donor strains, respectively. *Alphaproteobacteria* class was mainly represented by the families of *Rhizobiaceae*, *Kaistiaceae*, and *Beijerinckiaceae*, belonging to the *Rhizobiales* order. Other bacterial phyla, i.e., *Bacteroidota*, *Actinomycetota*, *Bacillota*, *Deinococcota*, and *Cyanobacteriota*, constituted a total of 8.1% (with *K. variicola* EEF15 donor) and 7.2% (with *E. coli* MG1655 donor) of the transconjugants. The bacterial community of lettuce rhizosphere showed the presence of the same bacterial families identified in conjugative experiments but at different relative abundance. For instance, some taxa were less abundant in the original rhizosphere bacterial community compared to the transconjugant pools, e.g., families *Pseudomonadaceae* and *Aeromonadaceae* that were, respectively, the 22 and 14% of the total ASVs, suggesting that these families are more prone to the acquisition of the conjugal broad host range plasmid compared to the rest of the community.

## 4 Discussion

Considering the reuse of treated wastewater as an irrigation source in the One Health context, *K. variicola* was chosen as a donor strain since indicated as an emerging pathogen for humans (Rodríguez-Medina et al., 2019). *K. variicola* (belonging to the *Klebsiella pneumoniae* complex in the *Enterobacteriaceae* family) has been reported to be associated with different environments, such as plant niches (e.g., rhizosphere), freshwater bodies, humans, insects, and other animals, as suggested by its name (the term “var*iicola*,” coming from Latin, indicates that the bacterium can be found in different places) (Rodríguez-Medina et al., 2019; Barrios-Camacho et al., 2019). In addition to its pathogenicity and wide distribution, it is intrinsically resistant to ampicillin (*bla*_LEN_), and reports on multidrug-resistant isolates of *K. variicola*, not only from clinical settings, are increasing (Barrios-Camacho et al., 2019; Rodríguez-Medina et al., 2019). For these reasons and considering the strain’s ability to colonize the lettuce rhizosphere (as shown in this study), where bacterial activity is enhanced if compared to bulk soil (Zhu et al., 2018), and to reach the endosphere, the strain *K. variicola* EEF15 has been selected as the perfect candidate to study conjugation events in complex plant-associated communities.

Gaining insight into plasmid dissemination when treated wastewater is reused as an irrigation source is of compelling interest. To this aim, the EEF15 donor strain of environmental origin (isolated from treated wastewater) was genetically manipulated to assess plasmid transfer to the complex natural bacterial community associated with the lettuce rhizosphere. Notably, plasmid conjugal transfer was specifically verified without the application of a selective agent (Klümper et al., 2015; Berthold et al., 2016; Fan et al., 2019; Xu et al., 2021). Our environmental donor strain was obtained following a strategy widely employed in previous studies (Klümper et al., 2015; Li et al., 2020): the selected strain was chromosomally tagged with a cassette carrying a red fluorescence protein (*mCherry*), a *lacI^q^* repressor gene, and transformed with a plasmid of interest encoding a *gfpmut3* gene downstream of a *LacI*-repressible promoter (Musovic et al., 2010), enabling the tracking of the plasmid movement to the recipient bacteria by flow cytometry. The following sorting of GFP-tagged cells and the application of 16S rRNA gene amplicon sequencing on the sorted cells allowed the identification of transconjugants (Klümper et al., 2014). This approach, based on cultivation-independent techniques, allowed to avoid the use of cultivation-based methods which are biased by the very low bacterial culturability in laboratory conditions (Joseph et al., 2003): estimates have indicated that only 1.4–14.1% of the total cells in a soil sample are indeed cultivable (Janssen et al., 2002).

In our study, we showed that the transfer of pKJK5::Plac::*gfp* plasmid occurred from the donor strain *K. variicola* EEF15::*lacI^q^-pLpp-mCherry-gm^R^* to both a selected recipient (i.e., a *P. putida* strain) and the complex bacterial community of plant rhizosphere, as confirmed by PCR and fluorescence (stereo)microscopy. We observed conjugal transfer to the rhizosphere community with a frequency of 10^−3^ with both donors. Lower values of conjugation frequency (i.e., 10^−4^) have been reported in the recent publication by Macedo et al. (2022) in which authors described the conjugal transfer of the plasmid pKJK5 in soil microcosms with or without manure. However, considering the diversity of the experimental set-ups (microcosms vs. filter mating assays, as well as differences in cell density, temperature, contact period, and nutrient availability), comparisons among studies should be made with caution (He et al., 2024; Pallares-Vega et al., 2021). Moreover, it is noteworthy to mention that, even if we observed a statistically significantly lower conjugal transfer rate with the environmental *K. variicola* donor strain than with the laboratory *E. coli* one, EEF15- and MG1655-derived pools of ASV transconjugants shared the same taxonomy and taxonomic composition (Figures 2, 3). A lower frequency of plasmid transfer by an environmental isolate cannot, nevertheless, be considered a lower risk for ARG circulation. Finally, we need to consider that the use of laboratory strains could not provide an exhaustive estimation of the impact of conjugal transfer since they might have lost important functions or regulatory genes that might be essential in “real-world” situations, e.g., proteins related to the osmotic stress or genes involved in cell to cell aggregation and biofilm generation (Sim et al., 2014; Tchagang et al., 2018), in comparison to environmental strains, which are adapted to thrive in specific habitats (Fux et al., 2005). Since their first isolation, researchers have sub-cultured laboratory strains such as *E. coli* K12 (from which derives the laboratory donor strain MG1655, used in this study) or *P. aeruginosa* PAO1 for many years, likely differentiating new phenotypes (Fux et al., 2005). For instance, if we compare uropathogenic and enteropathogenic strains to the avirulent laboratory strain of *E. coli* K12 through genomics analysis, only 39% of their combined set of proteins are shared (Welch et al., 2002; Fux et al., 2005). Future studies should aim to obtain a better comprehension of the magnitude of the phenomenon, and this should not disregard the use of environmental donor strains and experimental settings closer to real environmental situations. The use of environmentally relevant strains, which could own some specific traits influencing HGT outside of laboratory conditions, should help to disentangle the significance and mechanisms of AMR diffusion in different ecosystems, providing a more reliable description of AMR spread (Bernardy et al., 2016; Pinilla-Redondo et al., 2018; Fan et al., 2019; Riva F. et al., 2020; Riva V. et al., 2020; Di Cesare et al., 2022).

Considering the identification of taxa of the bacterial community associated with the lettuce rhizosphere having the potential to acquire exogenous plasmids by a conjugative donor, the phylum *Pseudomonadota* with the class *Gammaproteobacteria* was the dominant one, as reported in other soil-related studies on HGT evaluation (Klümper et al., 2015; Fan et al., 2019). However, Klümper et al. (2015) studied the conjugal transfer between several donors and the bacterial community extracted from the CRUCIAL soil, which was indeed the same soil that we considered in our experiments but to which lettuce plants applied a selection, recruiting bacteria in the rhizosphere. As already reported, the broad host range plasmid pKJK5 (a plasmid belonging to the IncP-1ε subgroup) was transferred to recipients belonging to different bacterial phyla in addition to *Pseudomonadota* (Klümper et al., 2015), i.e., *Actinomycetota* and *Bacteroidota*, for some of which poor culturability has been reported (Joseph et al., 2003): conjugation events between the Gram-negative donors and Gram-positive recipient bacteria have been indeed detected in both transconjugant pools, confirming what already documented in other studies, about the plasmid transmissibility to a broad phylogenetic range (Klümper et al., 2015; Fan et al., 2019; Qiu et al., 2018). Contrary to Klümper et al. (2015), we did not detect any differences between the transconjugant pools obtained from the two donor strains in terms of ASV taxonomy and taxonomic composition, indicating that in our study, the broad host range plasmid pKJK5, independently from the donor strain considered, could be acquired by the same bacterial taxa even if with a different conjugal frequency. However, the reasons behind the plasmid ability to be transferred to the same taxa are unclear (Klümper et al., 2015): we hypothesized that EEF15 and MG1655 donor strains, even if belonging to different species within the *Enterobacteriaceae* family, shared some characteristics which could favor cell-to-cell contact at the same extent, e.g., mating pair stabilization (Hirt et al., 2002; Virolle et al., 2020; Low et al., 2022). Moreover, the method we applied cannot allow us to discriminate between the horizontal plasmid transfer and the subsequent vertical transmission due to the growth of transconjugants on filter mating plates incubated at 25°C for 48 h (Klümper et al., 2015): the relative abundance of transconjugant taxa could be thus influenced by their growth rates, especially when comparisons with the taxa relative abundance of native rhizosphere are made. Finally, as also indicated by Klümper et al. (2015), the use of a Nycodenz-based extraction protocol resulted in the selection of a part of the bacterial microbiome associated with the lettuce rhizosphere, which was further exposed to plasmid transfer experiments: although we were not able to recover all the rhizospheric bacteria, it is noteworthy to mention that the same bias is applied with both donors; this could also allow to speculate that conjugation rates might be even higher in natural settings.

Among transconjugants, several bacterial families known to be able to thrive in the agri-food ecosystem, e.g., in plant and water-related microbiomes, as well as putative pathogenic bacteria derived from anthropogenic environments, have been detected. The best example is given by the most abundant family represented by *Pseudomonadaceae* with the genus *Pseudomonas*, known to be part of the plant and soil microbial communities, to be capable of suppressing plant pathogens, but also including possible pathogenic species/strains (Lyng and Kovács, 2023; Vieira et al., 2024; Dsouza et al., 2024). We retrieved several families (e.g., *Comamonadaceae*, *Xanthomonadaceae, Enterobacteriaceae*, *Rhizobiaceae*, and *Acetobacteraceae*) which are commonly retrieved in the plant microbiome (Riva et al., 2022b) and are known to be involved in plant physiology and fitness, i.e., including plant growth promoting rhizobacteria, bacterial taxa able to limit the growth of microbial phytopathogens, and plant pathogens (Vicente et al., 2023; Kaur et al., 2023; Bhadrecha et al., 2023; Leach et al., 2017). Likely, among the most abundant transconjugants there were also several families primarily linked to AMR spread in aquatic environments, such as *Aeromonadaceae* and *Legionellaceae* (Suyamud et al., 2024; Falkinham, 2020), and *Flavobacteriaceae* and *Enterobacteriaceae* families known to be hosted by aquatic animal species and plants (Burbick et al., 2023). Finally, we observed the presence of families typically associated with humans, able to thrive in anthropized environments, such as *Streptococcaceae* and *Staphylococcaceae* (Ziarati et al., 2022; Lawhon et al., 2023). This highlights that the transfer of ARG-carrying plasmids can occur among bacteria relevant in the agri-food ecosystem, possibly contributing to the spread of ARGs to pathogens in human-related habitats.

## 5 Conclusion

In this study, we have evaluated the ability of an environmental donor strain, isolated from treated wastewater and genetically manipulated, to transfer a conjugal broad host range plasmid to the rhizospheric microbial community of lettuce plants grown in agricultural soil to assess ARGs spread. Plasmid transfer was demonstrated to occur in a wide phylogenetic group of the rhizosphere microbiome without any selective pressure. This, together with the donor strain’s ability to colonize the plant rhizosphere and to reach the leaf endosphere, highlights the importance of studying the magnitude of the diffusion of AMR determinants in the agri-food ecosystem, particularly related to vegetables eaten raw, which have the potential to become in direct contact with the human microbiome. Future studies taking advantage of environmental donor strains could allow us to obtain a better comprehension of AMR spread under the context of the One Health approach to appraise possible risks related to human health.

## Data Availability

The datasets presented in this study can be found in online repositories. The names of the repository/repositories and accession number(s) can be found in the article/Supplementary material.

## Footnotes

^1^https://www.ncbi.nlm.nih.gov/tools/primer-blast/

^2^https://posit.co/download/rstudio-desktop/
