# Supplementary material for: Conjugal plasmid transfer in the plant rhizosphere in the One Health context
Source: Front Microbiol. 2024 Aug 29;15:1457854. doi: 10.3389/fmicb.2024.1457854 (PMC11390587; doi:10.3389/fmicb.2024.1457854)
Supplement: Supplementary file 1 [file Data_Sheet_1.PDF]

Supplementary Material

Conjugal plasmid transfer in the plant rhizosphere in the One Health context

Francesco Riva[1], Arnaud Dechesne[2,†], Ester M. Eckert[3,4], Valentina Riva[1], Sara Borin[1], Francesca Mapelli[1], Barth F. Smets[2,5], Elena Crotti[1*]

[1]Department of Food, Environmental and Nutritional Sciences (DeFENS), University of Milan, Milan, Italy

[2]Department of Environmental Engineering and Resource Engineering, Technical University of Denmark, Kgs. Lyngby, Denmark

[3]CNR – IRSA Water Research Institute, Molecular Ecology Group (MEG), Verbania, Italy

[4]National Biodiversity Future Center, NBFC, Piazza Marina 61, 90133 Palermo, Italy

[5]Department of Biological and Chemical Engineering, Center for Water Technology, Aarhus Unmiersity, Aarhus DK

†current address: Department of Biotechnology and Biomedicine, Technical University of Denmark, Kgs. Lyngby, Denmark

*** Correspondence:**
Elena Crotti
elena.crotti@unimi.it

SUPPLEMENTARY TABLES

Supplementary Table 1. Donor strains used in the study.

Donor strain	Plasmid	Reference
Klebsiella variicola EEF15::*lacI^q^-pLpp-mCherry-gm^R^*	pKJK5::Plac::*gfp*	This study
Escherichia coli MG1655::*lacI^q^-pLpp-mCherry-km^R^*	pKJK5::Plac::*gfp*	Klümper et al. (2015)

Supplementary Table 2. Lettuce colonization by strain *K. variicola* EEF15 RIF-R when lettuce was bacterized with 10^8 and 10^9 cell/gram of soil (indicated as “EEF15 10^8 cell/grams of soil” and “EEF15 10^9 cell/grams of soil” in the names of the column). EEF15 RIF-R abundance in the rhizosphere and in the leaf endosphere is indicated as CFU per gram of soil or leaf tissue, respectively. Supplementary Table 2 is available in the Dataverse repository (https://doi.org/10.13130/RD_UNIMI/TMYYLT).

Supplementary Table 3. Conjugal transfer frequency detected by fluorescence stereomicroscopy. Twenty pictures (twenty-one for MG1655-filter 3) were taken for each filter. The number of transconjugants were counted as per Klümper et al. (2014). Conjugal transfer rate was computed by the following formula: (Transconjugants per pictures × filter area (μm^2))/((picture area (μm^2) × (recipients introduced originally)).

Next page

Klebsiella variicola EEF15 donor - filters																						
picture	1	2	3	4	5	6	7	8	9	10	11	12	13	14	15	16	17	18	19	20	21	Per Pictures
**Filter 1**	96	100	81	99	95	83	88	102	87	99	97	87	108	93	98	113	122	109	23	15	-	1795
**Filter 2**	88	79	99	99	99	101	43	93	43	73	100	72	125	77	76	72	79	122	128	93	-	1761
**Filter 3**	48	66	61	71	4	23	17	95	76	37	85	67	92	50	65	68	72	78	61	68	-	1204
**Filter 4**	114	138	115	150	141	157	139	84	151	75	124	112	107	129	52	67	97	73	75	112	-	2212
***Escherichia coli* MG1655 donor - filters**																						
picture	1	2	3	4	5	6	7	8	9	10	11	12	13	14	15	16	17	18	19	20	21	Per Pictures
**Filter 1**	130	182	176	196	131	103	133	74	56	109	103	84	114	95	144	90	115	122	129	103	-	2389
**Filter 2**	90	144	165	138	160	158	101	274	196	116	146	72	141	133	115	131	136	186	71	-	-	2673
**Filter 3**	97	120	101	76	108	108	157	83	110	128	199	81	199	119	120	166	99	124	90	95	159	2539
**Filter 4**	99	89	164	95	132	157	108	119	159	120	128	172	88	172	133	124	90	106	155	137	-	2547

Supplementary Table 4. ASV table related to the analysed samples: A) ASV taxonomical affiliation is expressed at the genus level; B) ASV table is analysed at the family level; C) Explanation of the sample names in A and B. Supplementary Table 4 is available in the Dataverse repository (https://doi.org/10.13130/RD_UNIMI/TMYYLT).

Supplementary Table 5. Alpha diversity indices (Richness, Shannon and Simpson) for each treatment: i) transconjugants obtained using *K. variicola* EEF15 donor strain; and ii) transconjugants obtained using *E. coli* MG1655 donor strain.

	Richness (n° of ASVs)	Shannon	Simpson
Klebsiella variicola EFF15 donor strain	267.25 ± 15.22	3.512 ± 0.241	0.892 ± 0.020
Escherichia coli MG1655 donor strain	252.75 ± 21.47	3.316 ± 0.430	0.876 ± 0.037

SUPPLEMENTARY FIGURES

Supplementary Figure 1. Schematic view of the experimental setup used in the study. Lettuce rhizosphere was extracted by density gradient and exposed to the red-tagged donor strains, carrying a *gfp* gene on a plasmid, the expression of which was under the control of *lacI^q^* gene carried on the chromosome. Taking advantage of the Gfp expression, transconjugant cells were sorted and identified through 16S rRNA gene amplicon sequencing. Picture was created in BioRender.com.

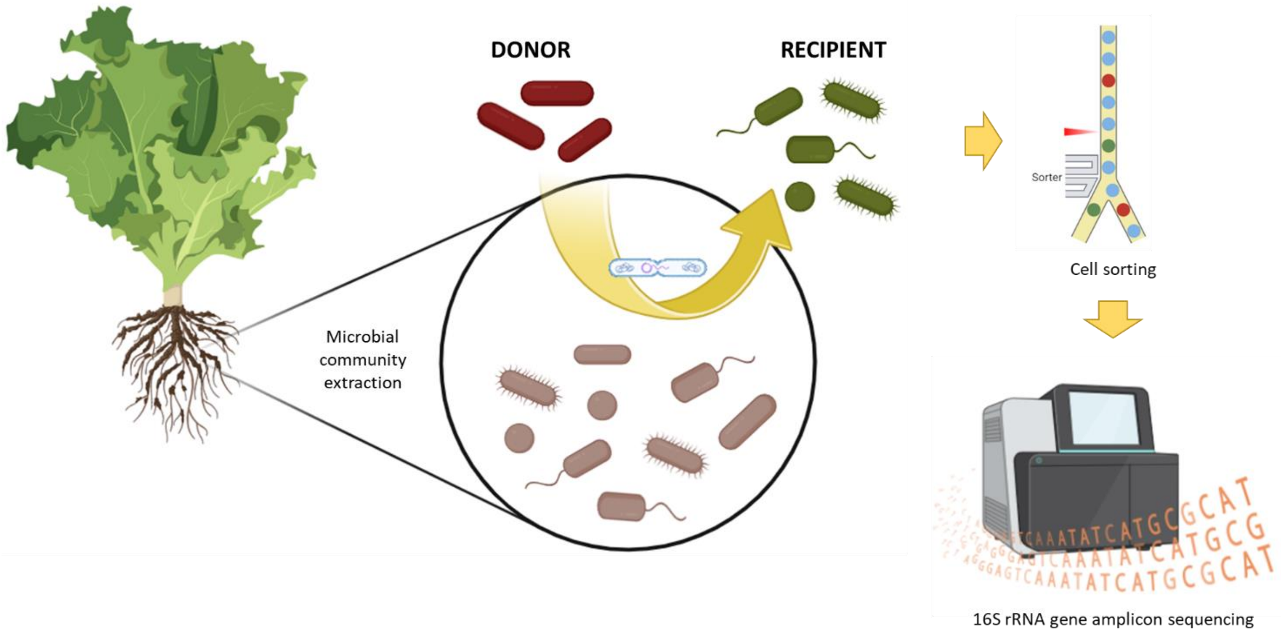

Supplementary Figure 2. Images of agarose gel electrophoresis of ITS-PCR amplicons of bacterial colonies isolated from lettuce rhizosphere (A) or lettuce leaf endosphere (B). (A) M: marker; PC: EEF15 RIF-R positive control; 1-18: random bacterial colonies isolated from the rhizosphere of lettuce plants bacterized with EEF15 RIF-R strain. (B) M: marker; PC: EEF15 RIF-R positive control; 1-12: random bacterial colonies isolated from endosphere of lettuce plants bacterized with EEF15 RIF-R strain.

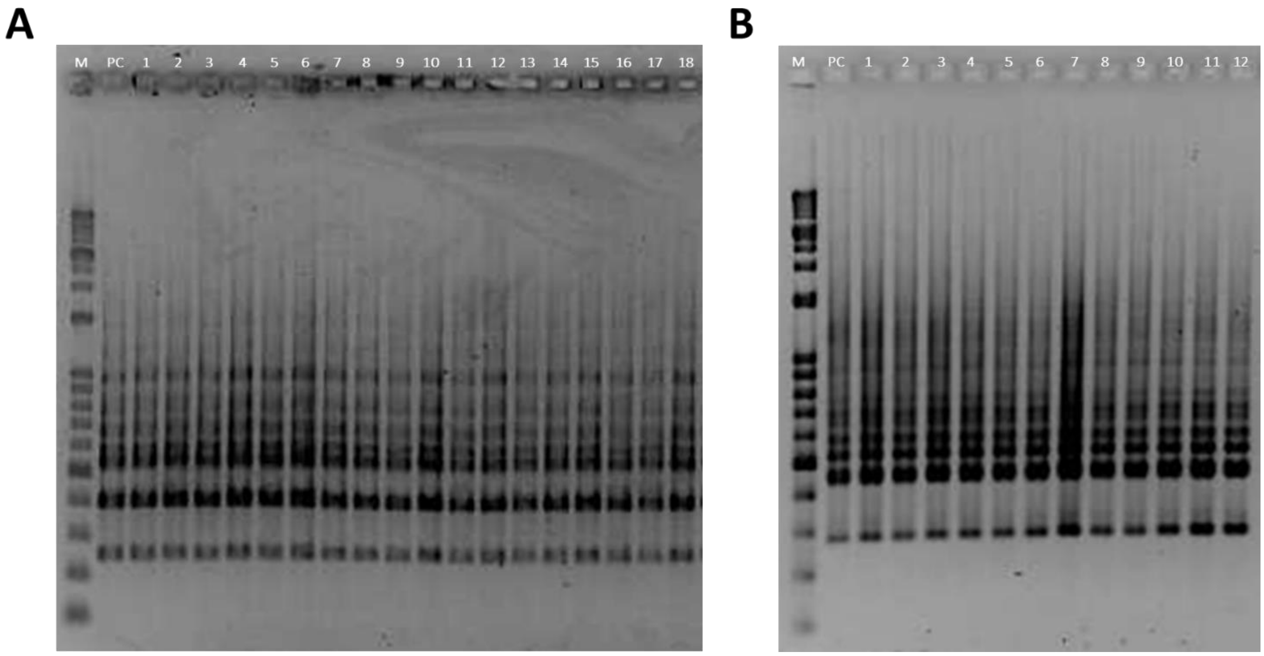

Supplementary Figure 3. Verification of filter mating conjugal transfer. (A) Images of agarose gel electrophoresis of Gfp-PCR amplicons of bacterial tranconjugants obtained in filter mating experiments between EEF15::*lacI*^{q}*-pLpp-mCherry-gm*^{R} pKJK5::Plac::*gfp* and *P. putida* KT2440. M: marker; PC: Gfp positive control; 1-9: random transconjugant isolates; NC: negative control. (B) Fluorescence microscopy image of a *P. putida* KT2440 transconjugant. Bar= 3.4 µm. (C) Image of a portion of the filter used in mating assays between strain EEF15::*lacI*^{q}*-pLpp-mCherry-gm*^{R} pKJK5::Plac::*gfp* and the lettuce rhizosphere community, visualized by fluorescence stereomicroscopy (picture area 0.6 mm^{2}). Green spots are transconjugants which received pKJK5::Plac::*gfp* plasmid, visualized trough *gfp* expression, while donor strain are observable as red spots due to *mCherry* expression.

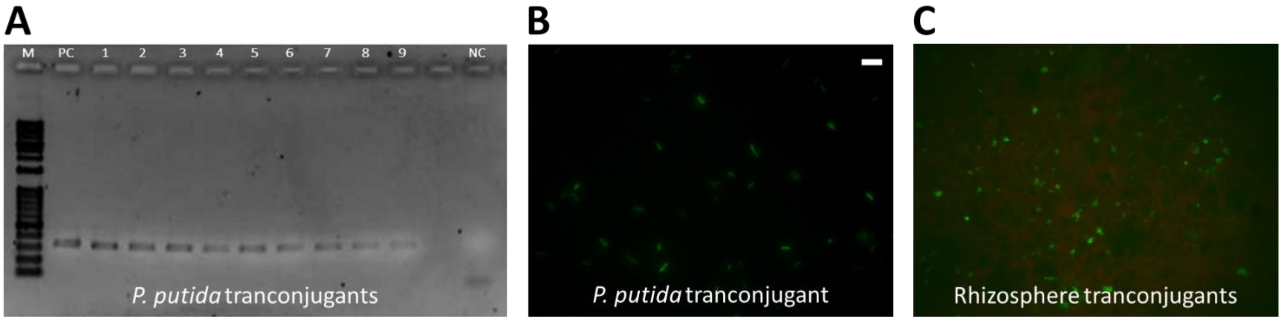

Supplementary Figure 4. Rarefaction curve of the 16S rRNA gene amplicon libraries evaluated for each sample.

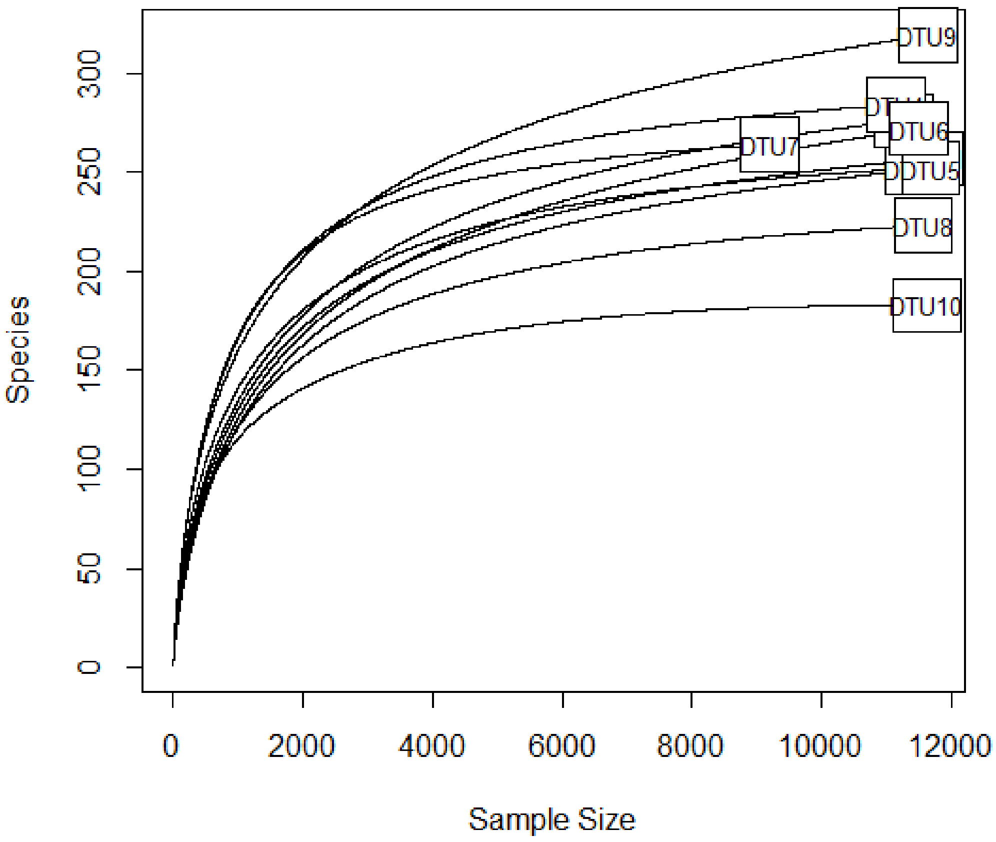

Supplementary Figure 5. Relative abundance of ASVs related to filter mating assays or from the original rhizosphere community (higher than 0.05% for at least one treatment) at family level. Eighty-two ASVs, which relative abundance is lower than 0.05%, are classified as “Others”. From the left to the right, histograms refer to transconjugants obtained in 4 replicate experiments with *Klebsiella variicola* EEF15::*lacI^q-pLpp-mCherry-gm^R* and *E. coli* MG1655::*lacI^q-pLpp-mCherry-km^R* donor strains, respectively. Last two histograms show the relative abundance of bacterial families of the community associated to lettuce rhizosphere.

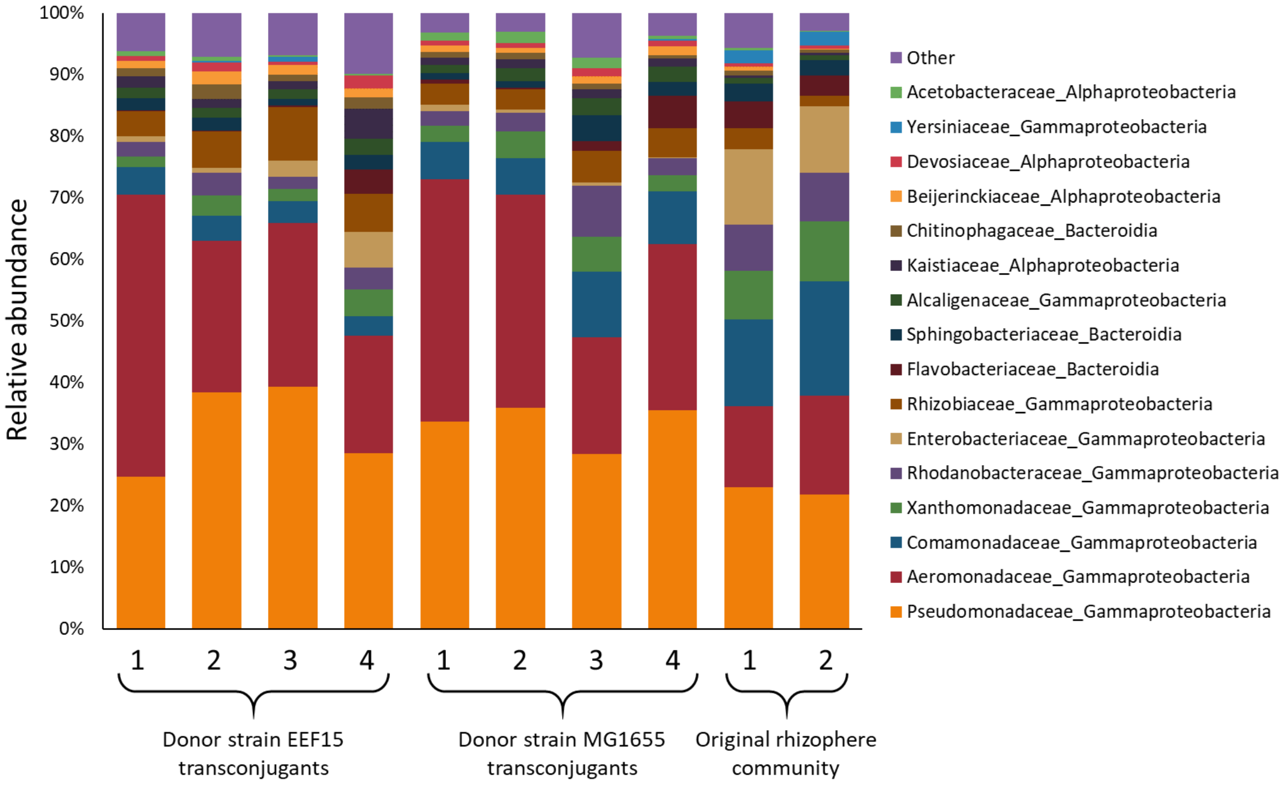

References

Klümper, U., Dechesne, A., & Smets, B.F. (2014). Protocol for evaluating the permissiveness of bacterial communities toward conjugal plasmids by quantification and isolation of transconjugants. In *Hydrocarbon and Lipid Microbiology Protocols* (pp. 275-288). Springer, Berlin, Heidelberg.

Klümper, U., Riber, L., Dechesne, A., Sannazzarro, A., Hansen, L. H., Sørensen, S. J., and Smets, B. F. (2015). Broad host range plasmids can invade an unexpectedly diverse fraction of a soil bacterial community. *ISME J*. 9(4), 934-945. doi:10.1038/ismej.2014.191
